# Reduced metal nanocatalysts for selective electrochemical hydrogenation of biomass-derived 5-(hydroxymethyl)furfural to 2,5-bis(hydroxymethyl)furan in ambient conditions

**DOI:** 10.3389/fchem.2023.1200469

**Published:** 2023-06-20

**Authors:** Baleeswaraiah Muchharla, Moumita Dikshit, Ujjwal Pokharel, Ravindranath Garimella, Adetayo Adedeji, Kapil Kumar, Wei Cao, Hani Elsayed-Ali, Kishor Kumar Sadasivuni, Naif Abdullah Al-Dhabi, Sandeep Kumar, Bijandra Kumar

**Affiliations:** ^1^ Department of Mathematics, Computer Science and Engineering Technology, Elizabeth City State University, Elizabeth City, NC, United States; ^2^ Laboratory of Environmental Sustainability and Energy Research (LESER), National Institute of Technology Delhi, New Delhi, India; ^3^ Biomass Research Laboratory (BRL), Old Dominion University, Norfolk, VA, United States; ^4^ Department of Natural Sciences, Elizabeth City State University, Elizabeth City, NC, United States; ^5^ Department of Electrical and Computer Engineering, Old Dominion University, Norfolk, VA, United States; ^6^ Center for Advanced Materials, Qatar University, Doha, Qatar; ^7^ Department of Botany and Microbiology, College of Science, King Saud University, Riyadh, Saudi Arabia

**Keywords:** electrochemical hydrogenation, 5-(hydroxymethyl)furfural (HMF), 2,5-bis(hydroxymethyl)furan (BHMF), nanocoral Ag, electrocatalysts, biomass

## Abstract

Selective electrochemical hydrogenation (ECH) of biomass-derived unsaturated organic molecules has enormous potential for sustainable chemical production. However, an efficient catalyst is essential to perform an ECH reaction consisting of superior product selectivity and a higher conversion rate. Here, we examined the ECH performance of reduced metal nanostructures, i.e., reduced Ag (rAg) and reduced copper (rCu) prepared via electrochemical or thermal oxidation and electrochemical reduction process, respectively. Surface morphological analysis suggests the formation of nanocoral and entangled nanowire structure formation for rAg and rCu catalysts. rCu exhibits a slight enhancement in ECH reaction performance in comparison to the pristine Cu. However, the rAg exhibits more than two times higher ECH performance without compromising the selectivity for 5-(HydroxyMethyl) Furfural (HMF) to 2,5-bis(HydroxyMethyl)-Furan (BHMF) formation in comparison to the Ag film. Moreover, a similar ECH current density was recorded at a reduced working potential of 220 mV for rAg. This high performance of rAg is attributed to the formation of new catalytically active sites during the Ag oxidation and reduction processes. This study demonstrates that rAg can potentially be used for the ECH process with minimum energy consumption and a higher production rate.

## Introduction

The rapid growth of the renewable electricity sector has created an unprecedented opportunity to electrify and enhance the sustainability of the primary production of a broad class of chemicals and materials (Schiffer and Manthiram, 2017; Luna et al., 2019). Many chemical transformations that are usually conducted at elevated temperatures and pressures can, in principle, be accomplished under much milder conditions, via electron-driven pathways (Kwon et al., 2013; Chatterjee et al., 2014; Li et al., 2014; Kwon et al., 2015; Schiffer and Manthiram, 2017; Chadderdon et al., 2019). At the same time, the production of chemicals, fuels, and solvents directly from biomass is of emerging interest to reduce the dependence on petroleum-based resources considering increasing environmental, economic, and political challenges (Kwon et al., 2016). The utilization of lignocellulose as a feedstock for fuels and chemicals can bring carbon neutrality, which implies reduced greenhouse gas emissions (Binder and Raines, 2009; Alonso et al., 2017; Preethi et al., 2021). There is a list of the top 10 renewable biomass-derived chemicals prepared by the Department of Energy in 2004. These chemicals are considered “platform chemicals” of the 21st century which can be used for producing bioproducts and biofuels. This will help in replacing fossil-derived chemicals and fuels. In 2010, this list was expanded to add 14 more platform chemicals including alcohols and sugars (ethanol, glycerol, sorbitol, and xylitol), acids (lactic, succinic, 3-hydroxypropanoic, and levulinic acid), furanics [furfural, HMF, and 2,5-furandicarboxylic acid (FDCA)], and biohydrocarbons (isoprene and others) (Bozell and Petersen, 2010; Galkin and Ananikov, 2019). Among others, furanics represent the most promising bioderived building blocks, 5-(Hydroxymethyl)furfural (HMF) has generally been accepted as a key element to bridge the gap from a fossil-based economy to a sustainable one (Chen et al., 2018). HMF is an important biomass platform compound, and it can be produced from cellulose that covers about 40% of lignocellulosic biomass (Kwon et al., 2016; Li et al., 2021). Numerous scientific groups are carrying out studies on the synthesis, and applications of HMF and its derivatives (Lewkowski, 2001; Binder and Raines, 2009; Kwon et al., 2013; Galkin and Ananikov, 2019; Li et al., 2021; Liu et al., 2021). HMF is referred to as a “sleeping giant” because of its enormous potential to bridge from fossil-based economy to chemistry to sustainable chemistry (Galkin and Ananikov, 2019). The difficulty in separating and purifying HMF from reaction media prevents the development of efficient HMF-based chemistry to this day (Hu et al., 2020; Li et al., 2022).

The presence of primary hydroxyl (-OH) and formyl (-C=O) in HMF makes it suitable for converting to various kinds of sustainable biochemicals (Xia et al., 2018; Mittal et al., 2020; Huang et al., 2022). One of the most important chemicals that could be produced by the selective hydrogenation of the formyl group of HMF is 2,5-bis(hydroxymethyl)-furan (BHMF) (Xia et al., 2018). The BHMF has applications in the synthesis of resins, polymers, artificial fibers, fuels, macrocycle polyethers compounds, and drugs (Chatterjee et al., 2014; Xia et al., 2020). In addition, BHMF can be further hydrogenated to 2,5-bis-(hydroxymethyl)-tetrahydrofuran (BHMTHF) which has applications in solvent, a monomer, and production of different high-value chemicals such as 1,6-hexanediol (Chatterjee et al., 2014). Generally, thermo-catalytic conversion routes in an aqueous medium have been attempted for converting HMF to BHMF using molecular hydrogen. Catalysts like Ni, Cu, platinum oxide (PtO), cobalt oxide (CoO) and molybdenum oxide (MoO) have been used for the reduction of the formyl group in HMF to produce BHMF (Lewkowski, 2001). Cu or Pt catalysts yielded 80%–100% of BHMF, while BHMTHF was the predominant product over Ni and Pd catalysts (Lewkowski, 2001; Chadderdon et al., 2019). Gold sub-nano clusters supported on alumina, Ru/CeO_2_, bimetallic catalysts (Ni-Pd), Ir-ReO_x_, and copper-doped porous metal oxide have also been used in thermo-catalytic conversion processes (Chatterjee et al., 2014). However, higher hydrogen pressure, high temperature, longer reaction time, and the use of organic solvents are essential components to achieve a high yield of BHMF (Chatterjee et al., 2014). With respect to the biochemical conversion route, catalytic synthesis of BHMF from HMF by recombinant *Saccharomyces cerevisiae* or a yeast strain-*Meyerozyma guilliermondii* was studied and a 99% selectivity and a 94% yield of BHMF within 24 h at the substrate concentration of 250 mM was achieved. However, HMF to BHMF biochemical conversion reaction is very sluggish (Xia et al., 2020) (Li et al., 2017).

**FIGURE 1:**
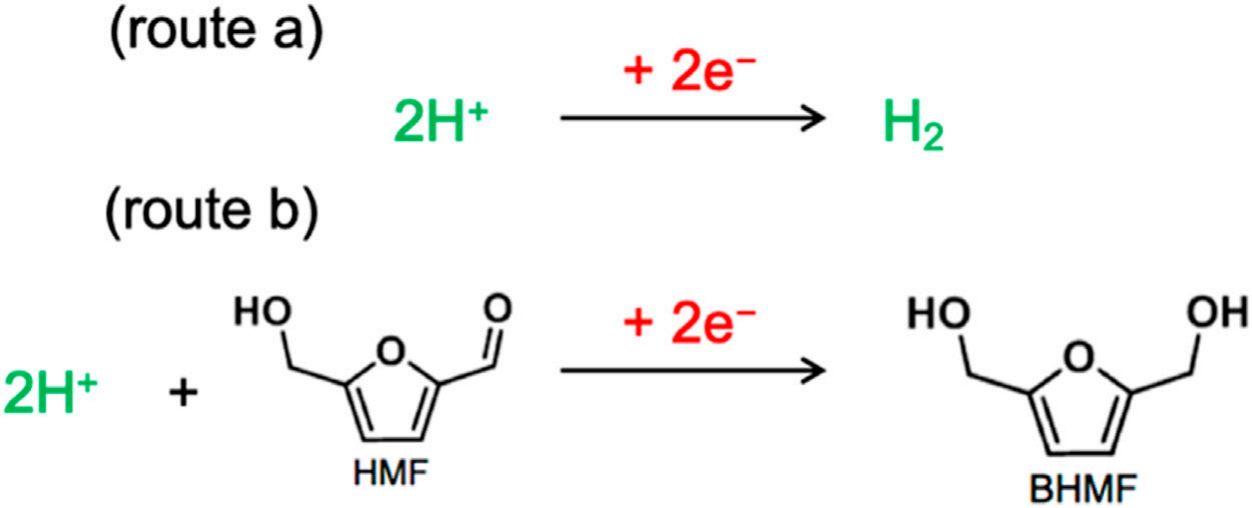
Schematic of ECH and HER: The protons can either produce hydrogen or it can be used for ECH reaction for HMF to BHMF formation (the simplest possible reaction pathway).

Electrochemical conversion route for HMF to BHMF is an environmentally benign method (Kwon et al., 2013; Nilges and Schröder, 2013; Kwon et al., 2015; Roylance et al., 2016; Li et al., 2017). The essential proton can be *in-situ* electrochemically generated by the electroreduction of proton or water which can be directly used to convert HMF into BHMF in place of hydrogen production (Kwon et al., 2015; Chen et al., 2018; May and Biddinger, 2020). This helps in avoiding the kinetic barrier for H_2_ activation which makes ECH to be performed at milder conditions. However, hydrogen evolution reaction (HER) possess a significant challenge and lowers the Faradaic efficiency of the ECH process (Chen et al., 2018) (Figure 1).

Kawana carried out the electrolysis of HMF and its derivatives at a platinum anode in methanol as a solvent with lithium perchlorate as a supporting electrolyte (Lewkowski, 2001). Kwon et al. (2013) investigated the ECH of HMF on a large number of metal cathodes (Fe, Ni, Ag, Zn, Cd, In, Pd, Al, Bi, Pb, Co, Au, Cu, Sn, and Sb) and concluded that BHMF was the major product over Fe, Ni, Ag, Zn, and Cd and in neutral conditions (0.1 M Na_2_SO_4_).

Oxide-derived nanomaterials consist of unique morphology and enhance catalytic activity due to the reduction of oxidized layer originating during the thermal or electrochemical oxidation processes (Li and Kanan, 2012; Li et al., 2014; Theaker et al., 2018; Hill-Dick et al., 2021). For example, Ag synthesized via electrochemical oxidation and reduction process, reduced Ag (rAg), and oxide derived Cu prepared via thermal oxidation and electrochemical reduction processes, rCu has shown excellent electrochemical catalytic activity for different electrochemical reactions including CO_2_ electrochemical conversion, glucose oxidation and others (Li and Kanan, 2012; Li et al., 2014; Theaker et al., 2018; Hill-Dick et al., 2021). Also, in the case of CO_2_ electrochemical reduction in aquas media, the competitive hydrogen evolution reaction was also suppressed in the presence of rAg and rCu catalysts (Li and Kanan, 2012; Li et al., 2014; Theaker et al., 2018). Thus, we hypothesize that reduced metal oxides such as rAg and rCu could be excellent catalysts for ECH of HMF in terms of overall performance including selectivity, rate of reaction, and overpotential. To examine this hypothesis, we synthesized and performed ECH of HMF in aqueous media using rAg and rCu as catalysts, and the results were compared with pristine Ag and Cu. As expected, rAg and rCu have improved ECH performance for HMF to BHMF conversion selectivity attributed to the higher surface area and origin of new catalytic active sites. Moreover, rAg exhibits relatively higher ECH performance in comparison to rCu due to its intrinsic catalytic properties.

## Results and discussion

### Surface analysis

**FIGURE 2:**
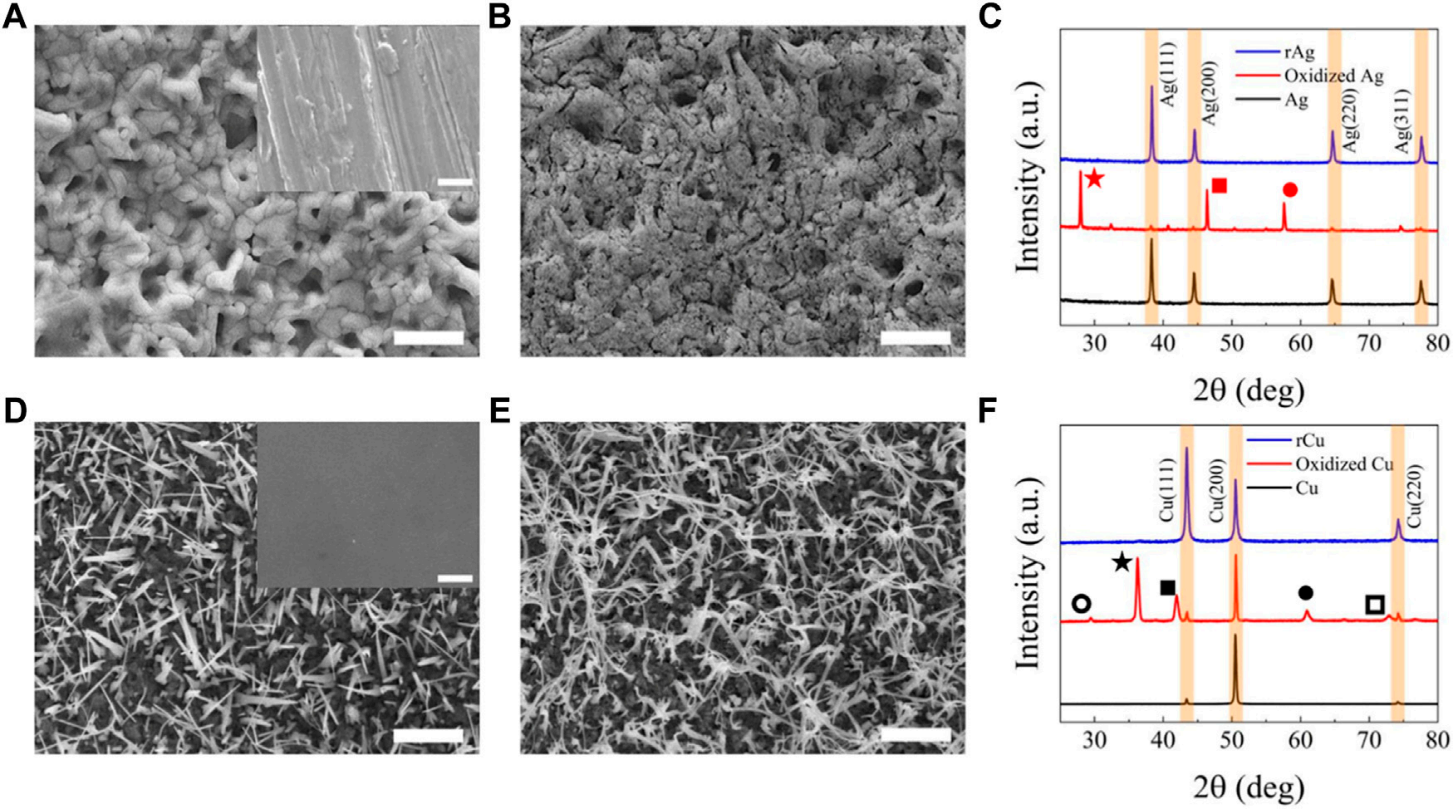
Physical analysis of catalysts: SEM image of (A) Ag after 12 h oxidation in 0.1 M KCl solution at 0.3 V versus RHE (in the inset pristine Ag) (B) Ag after reduction in 0.1 M KHCO_3_ solution at 0.3 V versus RHE, (C) XRD analysis of pristine Ag oxidized Ag [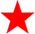
AgCl (111), 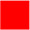
AgCl (220) and 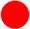
AgCl (222)] and reduced Ag, (D) Cu after thermal oxidation at 400°C for 1 h (in the inset pristine Cu), (E) Cu after electrochemical reduction in 0.1 M KHCO_3_ solution at 0.3 V versus RHE. The scale bar is 5 µm for **(A, B)** and 2.5 µm for **(D–F)** XRD analysis of pristine Cu, oxidized Cu [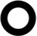 Cu_2_O (110) 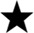
Cu_2_O (111) 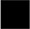 Cu_2_O (200) 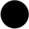 Cu_2_O (220) and 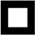 Cu_2_O (311)] and reduced Cu.

Figure 2 shows the evolution in surface morphology of Ag and Cu films during the oxidation and reduction processes. In the case of Ag, the film has scratches due to mechanical polishing, which converts into a porous surface after the electrochemical oxidation process (Figure 2; Supplementary Figure S1; Supplementary Table S1). As discussed in our previous report, this transition is attributed to the formation of a thick and dense AgCl layer due to the oxidation process into KCl solution (Hill-Dick et al., 2021). During the reduction process, this AgCl layer becomes more porous and creates a nanocoral-type morphology due to the decomposition of AgCl into Ag^+^ and Cl^−^ respectively. Here it should be noted that Cl^−^ can diffuse into the electrolyte (KHCO_3_ solution) while Ag^+^ is reduced onto the surface maintaining nanocoral morphology. The energy dispersive spectroscopy (EDS) and X-ray diffraction (XRD) analysis further confirmed the formation of AgCl during oxidation as the Ag to Cl ratio was identified as 1:1 (Supplementary Figure S2; Supplementary Table S2). EDS analysis of rAg sample is provided in supplementary information (Supplementary Figure S3; Supplementary Table S3). The XRD analysis further supports the formation of AgCl as additional strong peaks associated with the AgCl (111), (202), and (222) planes identified in Figure 2C. However, after the reduction process, we did not observe any Cl on the surface of the rAg film as evidenced by XRD analysis (Figure 2C). More importantly, we observed that the full width at half-maximum of the pristine Ag and rAg films are similar. This clearly indicates that the changes due to oxidation and reduction processes are morphological rather than crystallinity.

In the case of Cu, we thermally oxidized the Cu film at 400°C without using inert gases (e.g., N_2_ and Ar) (Li and Kanan, 2012; Li et al., 2014; Theaker et al., 2018). During oxidation at 400°C, we observed the formation of spikes due to fast oxidation kinetics. The SEM images collected after the electrochemical reduction process show the formation of dense nanofibril structures as a result of conversion of Cu oxides into Cu evidenced by corresponding XRD (Figures 2E, F). Further EDS and XRD analysis before and after oxidation and reduction processes suggest oxides formation and elimination during oxidation and reduction processes, respectively (Figure 2F; Supplementary Figures S4-S6; Supplementary Tables S4-S6).

### Electrochemical performance of oxide-derived reduced metals for HMF hydrogenation

**FIGURE 3:**
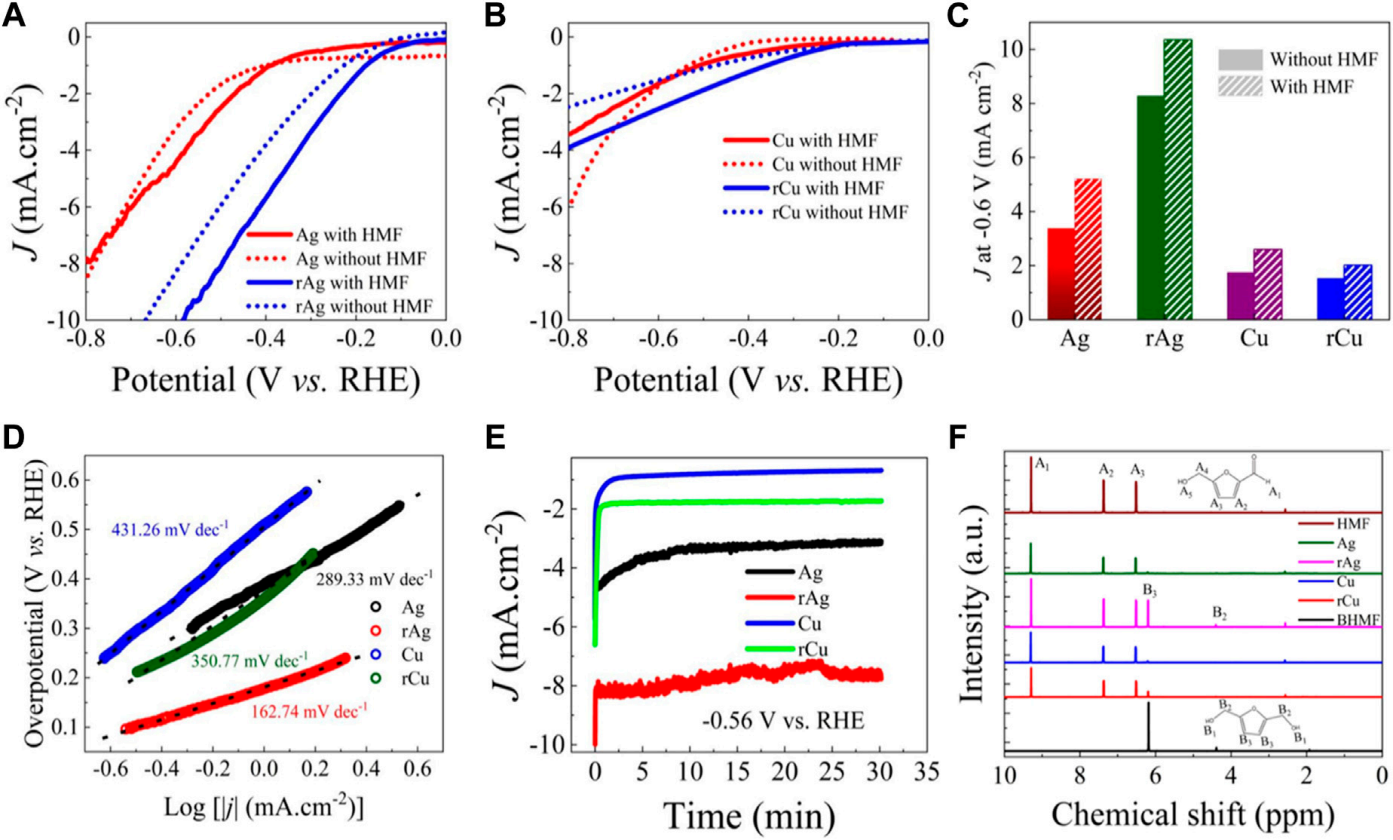
Electrochemical performance of the catalysts: LSV collected in buffer solution and buffer solution with 20 mM HMF solution for **(A)** Ag, rAg, **(B)** cu and rCu catalysts. **(C)** Comparison of current densities obtained at 0.6 V versus RHE for all catalysts in buffer and buffer with 20 mM HMF solution. **(D)** Tafel slope for Ag, rAg, Cu and rCu catalysts obtained from LSV collected in 20 mM HMF buffer solution. **(E)** CA experiments for Ag, rAg, Cu and rCu at 0.56 V versus RHE performed in 20 mM HMF buffer solution and **(F)** corresponding 1H NMR spectra for product identification.

The ECH of HMF by Ag, rAg, Cu, and rCu were investigated by performing CV with 20 mM HMF and without HMF buffer solution (borate buffer solution, pH—8) using two-compartments separated by frits three electrodes electrochemical cell. Initially, N_2_ was purged into the electrolyte (without HMF) and potential was scanned from 0 to 0.8 V versus RHE followed by similar experiments but in 20 mM HMF buffer solution as an electrolyte Figure 3A. Pristine Ag has shown similar *I-V* behavior in both scenarios as similar current densities were observed within the applied potential window (0 to −0.8 V versus RHE) Figure 3A. An 8 mA/cm^2^ current density was observed for Ag under both experimental conditions (with and without HMF) at −0.8 V versus RHE. Interestingly, in the case of rAg, the *I-V* curve positively shifts (∼200 mV) for both hydrogen evolution and ECH reactions. The rAg exhibits higher current densities for HMF hydrogenation and HER as well. A 10 mA/cm^2^ current density was recorded at −0.7 and −0.6 V versus RHE in buffer solution and buffer solution with 20 mM HMF. Here it should be noted that the higher surface area of rAg due to high porosity may result in higher HMF hydrogenation current density. However, the onset potential for HMF hydrogenation reaction on rAg (0.2 V versus RHE) is much lower in comparison to the Ag (0.5 V versus RHE) and independent of surface area. Similar results were reported for oxide-derived Ag in 0.5 M borate buffer (pH 9.2) with 98.2% selectivity of HMF to BHMF but with relatively poor current density (Supplementary Table S8) (Liu et al., 2021). These results indicate that oxidation/reduction processes introduce new types of catalytically active sites, enabling the reduction of the HMF hydrogenation reaction potential as the onset potential is less impacted by surface area.

Considering Cu-based catalysts, it shows relatively poor catalytic activity for HMF ECH reaction (Figure 3B). In buffer solution, the Cu film exhibits higher HER current density (-6 mA/cm^2^) in comparison to the rCu (−2.5 mA/cm^2^) at −0.8 V versus RHE. It might be due to the formation of a stable oxide layer during the thermal oxidation process as evidenced by XRD. However, in 20 mM HMF solution (−3.5 mA/cm^2^), we have noticed a decrease in the current density for Cu catalyst in comparison to the obtained in only buffer solution (−5.5 mA/cm^2^). This might be due to surface coverage by HMF molecule inhibiting HER and simultaneously promoting HMF ECH reaction. While this is not the case for rCu. The rCu shows a higher current density in HMF solution (−4 mA/cm^2^) in place of the current density recorded in buffer solution (−3.5 mA/cm^2^). This diverse behavior of rCu relative to pristine Cu might be attributed to the intrinsic activity of rCu due to the different nature of the surface in terms of morphology and crystallinity.

Here it should be noted that there are two possible HFM to BHMF hydrogenation pathways in basic or neutral media (Kwon et al., 2015). It can be either utilizing hydrogen from the water (HMF + 2H_2_O + 2e− → BHMF + 2OH−) or surface adsorbed hydrogen (H*) (HMF + 2H* → BHMF) (Kwon et al., 2015; Roylance et al., 2016; Chadderdon et al., 2019; Sanghez de Luna et al., 2021). The hydrogenation process where adsorbed hydrogen participates in the reaction is considered ECH. In the case of Cu- and Ag-based catalysts, ECH is considered the primary pathway for HMF to BHMF conversion.

Figure 3C represents the differences in current densities for all examined catalysts at −0.6 V versus RHE where rAg catalyst exhibits −10 mA/cm^2^ HMF hydrogenation current density. The rAg has 4, 5, and 3.3 times higher HMF ECH current density in comparison to the Ag, Cu, and rCu catalysts, respectively. The results indicate the superiority of rAg followed by rCu over other catalysts. The Tafel slope for all catalysts further provides key information about their activities (Figure 3D). The pristine Ag and Cu exhibit a Tafel slope of 289.33 mV/dec and 431.26 mV/dec, respectively. This contrasts with previously reported Tafel slopes for Ag and Cu in buffer solution with HMF where a lower Tafel slope was observed for Cu (∼54 mV/dec) in comparison to the Ag (∼73 mV/dec) (Sanghez de Luna et al., 2021). However, here it should be noted that the current data is not iR corrected while previously reported Tafel slopes were obtained for iR corrected data (Sanghez de Luna et al., 2021). Despite high Tafel slopes obtained for all samples, the Tafel slope is much lower for rAg (192.7 mV/dec) and rCu (350.77 mV/dec) indicating superior HMF hydrogenation activity Supplementary Table S7. The lower slope values for rAg and rCu suggest that the charge transfer for HMF reaction is much faster in comparison to their parent catalysts and superior HMF catalytic activity is not only attributed to the surface modification but also the origin of efficient catalytic active sites (Sanghez de Luna et al., 2021).

To confirm the onset potential of HMF hydrogenation, we also performed CA experiments at 0.56 V versus RHE for Ag, Cu, rAg, and rCu in 20 mM HMF solution for 30 min (Figure 3E). The collected catholyte solutions were analyzed using 1H NMR spectroscopy (Figure 3F) (Long et al., 2018). In Figure 3F, the NMR spectra of HMF and BHMF are clearly distinct from each other due to their chemical structure. The NMR spectra of 20 mM HMF/solution indicate the presence of three HMF characteristics peaks at 6.2, 7.4, and 9.3 ppm, respectively. The NMR spectra for electrolytes collected at 0.56 V versus RHE after 30 min on Ag and Cu are similar to 20 mM HMF/solution. These results confirm that the HMF hydrogenation reaction does not take place on Ag and Cu at this potential. On the other hand, three clear peaks at 2.5, 4.3, and 6.1 ppm were observed for electrolytes collected after 30 min electrochemical reaction on rAg and rCu at 0.56 V versus RHE. These peaks are associated with the BHMF and overlap with the NMR spectra collected for pure BHMF solution. The results confirm that rAg and rCu can catalyze HMF to BHMF hydrogenation reaction at the low potential in comparison to the pure Ag and Cu metal-based catalysts. Moreover, among all examined catalysts, rAg exhibits superior performance as evidenced by recording the highest current density and lowest onset potential among all examined catalysts under similar experimental conditions.

**FIGURE 4:**
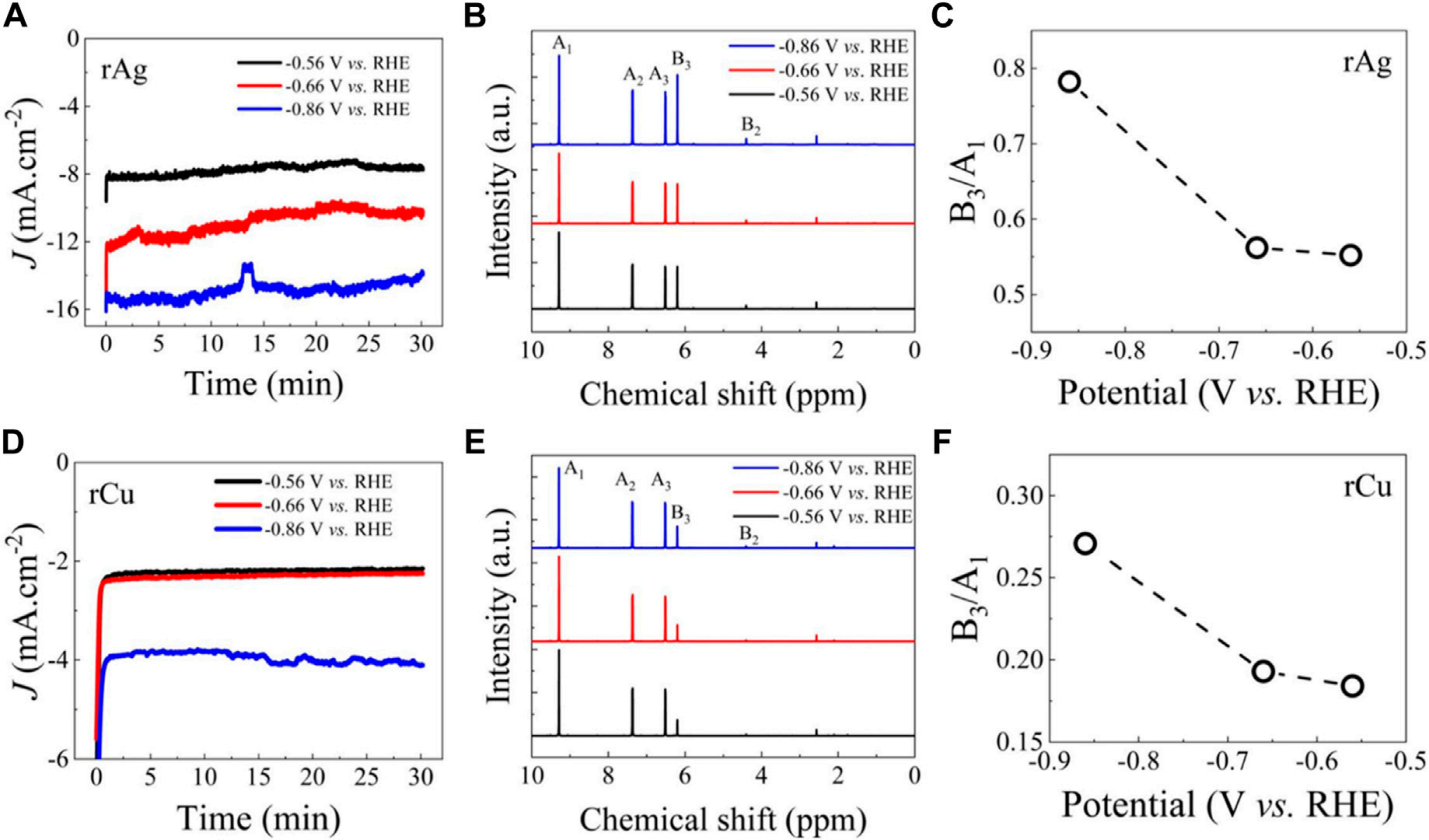
Comparative ECH performance of the rAg and rCu catalysts: **(A)** chronoamperometry (CA) results collected in buffer solution with 20 mM HMF solution at different potentials for rAg. **(B)** Corresponding NMR spectra for the collected electrolyte after 30 min CA experiments. **(C)** BHMF NMR spectra peak (B3) to HMF NMR spectra peak (A1) ratio. **(C)** CA results collected in buffer solution with 20 mM HMF solution at different potentials for rCu. **(D)** Corresponding NMR spectra for the collected electrolyte after 30 min CA experiments. **(E)** BHMF NMR spectra peak (B3) to HMF NMR spectra peak (A1) ratio. The results confirm higher ECH activity of rAg catalysts.

Next, we perform HMF hydrogenation for rAg and rCu at different potentials ranging from 0.56 to 0.86 V vs. RHE and analyze the BHMF product formation (Figure 4). After 30 min, the NMR of the catholyte was used for liquid phase product identification. Only BHMF was identified as the ECH main product, confirming the selectivity of rAg and rCu for HMF to BHMF production. BHMF concentration and yield were estimated from constant potential electrolysis (Supplementary Table S9). The intensity of the NMR peak associated with BHMF increases as higher potential was applied and attained maximum peak height for the sample collected at 0.86 V versus RHE for both catalysts. Even at this potential, no side product was identified confirming the 100% selectivity to BHMF formation of rAg and rCu even at a higher ECH rate. We also calculated the HMF and BHMF peak intensity ratio. BHMF concentration was estimated from NMR data (Supplementary Table S10), BHMF concentration is increasing with the increase in applied potential. As expected, the highest ratio of HMF characteristic peak (9.2 ppm) to BHMF characteristic peaks (6.2 ppm) was observed when the HMF hydrogenation reaction was performed at 0.86 V versus RHE confirming the higher HMF to BHMF conversion rate as expected due to higher current density.

## Conclusion

In summary, we have shown that rAg and rCu work efficiently for the ECH of HMF. Among both Ag and Cu-based catalysts, rAg exhibits the highest ECH performance which can be attributed to the higher surface area and intrinsic catalytic properties of the rAg catalysts. At the same working potential (e.g., 0.86 V versus RHE), rAg nanocatalysts exhibit approximately two times higher ECH reaction current density in comparison to the rCu catalyst resulting in higher BHMF formation. Our results indicate that oxide-derived nanocatalysts can be potentially used for selective HMF to BHMF electrochemical hydrogenation reaction, however, the ECH rate depends on the intrinsic properties of catalysts rather than higher surface area.

## Materials and methods

### Chemicals

Sodium hydroxide (NaOH, pellets) purchased from Millipore Corp. Uric acid (C_5_H_4_N_4_O_3_, 99%) and dopamine hydrochloride (C_8_H_11_NO_2_•HCl, 99%) purchased from Alfa Aesar. Potassium chloride (KCl) and potassium bicarbonate (KHCO_3_) purchased from Fisher Chemical.

### Preparation of samples

The rAg and rCu catalysts were prepared using a previously reported method (Theaker et al., 2018; Hill-Dick et al., 2021). The oxidation of Ag and Cu was performed via electrochemical and thermal oxidation processes respectively. In the first case, the chemically cleaned Ag film (rinsed in 2-propanol and water) was used as a working electrode along with Ag/AgCl and Pt wire reference and counter electrode respectively. The electrochemical oxidation was performed in 0.1 M KCl for 12 h at an applied potential of 0.3 V (Supplementary Figure S1). Samples were washed with distilled water and then reduced in 0.1 M KHCO_3_ for 45 min at an applied potential of −1.2 V. While rCu samples were prepared via thermal oxidation at 400°C for 1 h followed by an electrochemical reduction in 0.1 M KHCO_3_ solution at 1.2 V (See Supplementary Material). The electrochemical experiments were performed using SP-200 potentiostat purchased from BioLogic.

### Characterization

The morphological structure and elemental composition of the samples were characterized using SEM. Surface morphologies of three Ag samples were imaged using a JEOL JSM-6060LV SEM. Chemical analysis was carried out using a Thermo Scientific UltraDry EDS detector attached to the SEM. XRD analysis was conducted using a Rigaku MiniFlex II x-ray diffractometer. Supplementary Figure S2 shows the rough surface after the 12 h oxidation process.

### Electrochemical characterization

All electrochemical experiments were performed by using a typical two-compartment three-electrode electrochemical H-cell and a BioLogic potentiostat SP-300 (Supplementary Material). A borate buffer solution (500 mM, pH = 9.2) was prepared by mixing boric acid and sodium hydroxide in deionized water and was used as an electrolyte for all experiments. In this study, the prepared Ag, rAg, Cu, and rCu samples were used as working electrodes. A Pt mesh and Ag/AgCl saturated in KCl were used as counter and reference electrodes respectively. The cathode and anode compartments were separated via glass frit (pore size - 20 μm). HMF hydrogenation reaction kinetics were studied by performing linear sweep voltammetry measurements by sweeping the potential from 0.0 to 0.86 V versus RHE with the scan rate of 20 mV·s^−1^. All potentials were converted to the RHE using the Nernst equation, Potential vs. RHE = Applied potential versus Ag/AgCl (saturated KCl) + 0.197V + 0.0592 × pH.

The CA experiments were performed using 20 mM HMF solution (borate buffer solution, 9.2 pH) at constant potentials (0.56–0.86 V versus RHE) for 30 min. The collected samples were analyzed using NMR spectroscopy.

### NMR characterization

NMR experiments were carried out on a Bruker 400 MHz solution state spectrometer with z-gradients and with a broadband indirect detect probe in the COSMIC laboratory of Old Dominion University, using 10% D_2_O. The data were collected at room temperature, with a relaxation delay of 1.5 s, and with 256 scans. One-dimensional 1H spectra were acquired using a water suppression pulse sequence, PEW5shapepr (a modified Perfect Echo WATERGATE W5 sequence) with a train of water-selective shaped pulses applied during relaxation delay.

## Data Availability

The original contributions presented in the study are included in the article/Supplementary Material, further inquiries can be directed to the corresponding author.
