# Supplementary material for: Reduced metal nanocatalysts for selective electrochemical hydrogenation of biomass-derived 5-(hydroxymethyl)furfural to 2,5-bis(hydroxymethyl)furan in ambient conditions
Source: Front Chem. 2023 Jun 20;11:1200469. doi: 10.3389/fchem.2023.1200469 (PMC10318534; doi:10.3389/fchem.2023.1200469)
Supplement: Supplementary file 1 [file DataSheet1.docx]

**Supplementary File**

**Reduced Metal Nanocatalysts for Selective Electrochemical Hydrogenation of Biomass-Derived 5-(Hydroxymethyl)furfural to 2,5-bis(Hydroxymethyl)furan in Ambient Conditions**

Baleeswaraiah Muchharla^1^, Moumita Dikshit^2^, Ujjwal Pokharel^3^, Ravindranath Garimella^3^, Adetayo Adedeji^4^, Kapil Kumar^2^, Wei Cao^5^, Hani Elsayed–Ali^5^, Kishor Kumar Sadasivuni^6^, Naif Abdullah Al-Dhabi^7^, Sandeep Kumar^3^, and Bijandra Kumar^1*^

^1^ Department of Mathematics, Computer Science and Engineering Technology, Elizabeth City State University, Elizabeth City, NC 27909 USA.

^2^ Laboratory of Environmental Sustainability and Energy Research (LESER), National Institute of Technology Delhi, New Delhi 110040, India

^3^Biomass Research Laboratory (BRL), Old Dominion University, Norfolk, VA 23508, USA.

^4^Department of Natural Sciences, Elizabeth City State University, Elizabeth City, NC 27909 USA.

^5^Department of Electrical and Computer Engineering, Old Dominion University, Norfolk, VA 23529, USA.

^6^Center for Advanced Materials, Qatar University, Doha 2713, Qatar.

^7^Department of Botany and Microbiology, College of Science, King Saud University, Riyadh 11451, Saudi Arabia

**Fig. S1.** Energy dispersive spectroscopy (EDS) spectra of pristine Ag electrode.

**Table S1.** Quantitative EDS results of pristine Ag electrode.

| ***Element*** | ***Net***  ***Counts*** | ***Int.***  ***Cps/nA*** | ***Weight %*** | ***Weight %***  ***Error*** | ***Atom %*** | ***Atom %***  ***Error*** |
| --- | --- | --- | --- | --- | --- | --- |
| ***C*** | 1317 | --- | 2.02 | ± 0.05 | 15.64 | ± 0.40 |
| ***Ag*** | 57486 | --- | 97.98 | ± 0.96 | 84.36 | ± 0.82 |
| ***Ag*** | 0 | --- | --- | --- | --- | --- |
| ***Total*** |  |  | 100.00 |  | 100.00 |  |

**Fig. S2.** EDS spectra of Ag electrode electrochemically oxidized for 12 hours.

**Table S2.** Quantitative EDS results of Ag electrode electrochemically oxidized for 12 hours.

| ***Element*** | ***Net***  ***Counts*** | ***Int.***  ***Cps/nA*** | ***Weight %*** | ***Weight %***  ***Error*** | ***Atom %*** | ***Atom %***  ***Error*** |
| --- | --- | --- | --- | --- | --- | --- |
| ***C*** | 805 | --- | 4.09 | ± 0.12 | 15.89 | ± 0.47 |
| ***O*** | 713 | --- | 8.87 | ± 0.53 | 25.84 | ± 1.56 |
| ***Cl*** | 22314 | --- | 21.04 | ± 0.24 | 27.67 | ± 0.32 |
| ***Cl*** | 0 | --- | --- | --- | --- | --- |
| ***K*** | 2278 | --- | 2.72 | ± 0.26 | 3.25 | ± 0.31 |
| ***K*** | 0 | --- | --- | --- | --- | --- |
| ***Ag*** | 32494 | --- | 63.28 | ± 1.08 | 27.35 | ± 0.47 |
| ***Ag*** | 0 | --- | --- | --- | --- | --- |
| ***Total*** |  |  | 100.00 |  | 100.00 |  |

**Fig. S3.** EDS spectra of electrochemically reduced Ag electrode.

**Table S3.** Quantitative EDS results of reduced Ag electrode.

| ***Element*** | ***Net***  ***Counts*** | ***Int.***  ***Cps/nA*** | ***Weight %*** | ***Weight %***  ***Error*** | ***Atom %*** | ***Atom %***  ***Error*** |
| --- | --- | --- | --- | --- | --- | --- |
| ***C*** | 1125 | --- | 1.86 | ± 0.05 | 14.55 | ± 0.41 |
| ***Ag*** | 53480 | --- | 98.14 | ± 0.99 | 85.45 | ± 0.86 |
| ***Ag*** | 0 | --- | --- | --- | --- | --- |
| ***Total*** |  |  | 100.00 |  | 100.00 |  |


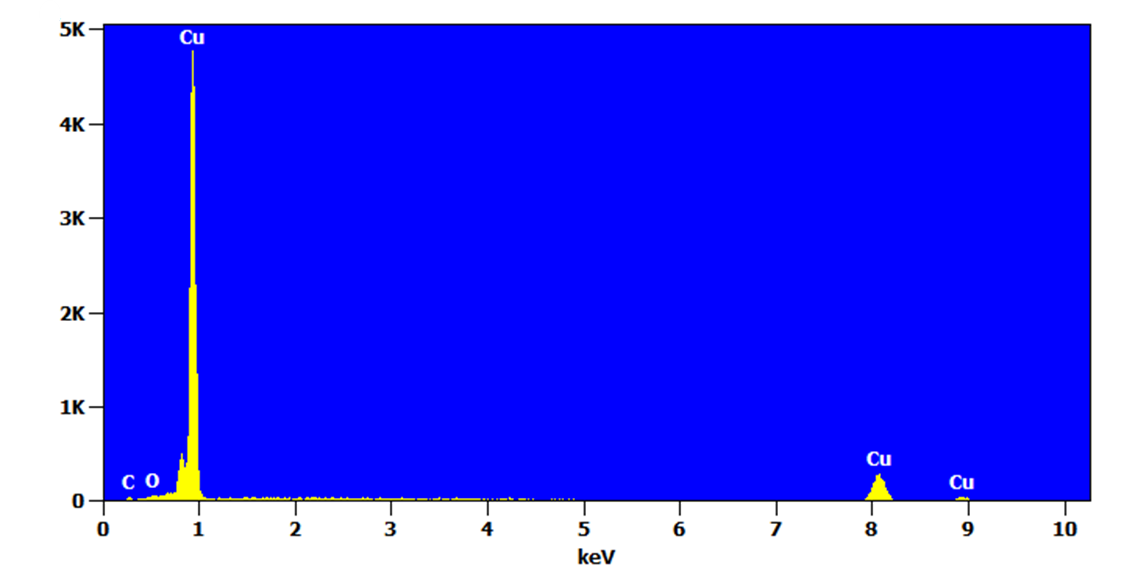


**Fig. S4.** EDS spectra of a cleaned Cu electrode

**Table S4.** EDS data of a cleaned Cu electrode

| ***Element*** | ***Net***  ***Counts*** | ***Int.***  ***Cps/nA*** | ***Weight %*** | ***Weight %***  ***Error*** | ***Norm.***  ***Wt.%*** | ***Atom %*** | ***Atom %***  ***Error*** | ***Compnd %*** |
| --- | --- | --- | --- | --- | --- | --- | --- | --- |
| ***C*** | 225 | --- | 2.03 | ± 0.14 | 2.03 | 9.74 | ± 0.65 | 2.03 |
| ***O*** | 100 | --- | 0.58 | ± 0.16 | 0.58 | 2.09 | ± 0.59 | 0.58 |
| ***Cu*** | 4760 | --- | 97.38 | ± 2.78 | 97.38 | 88.17 | ± 2.52 | 97.38 |
| ***Cu*** | 33624 | --- | --- | --- | --- | --- | --- | --- |
| ***Total*** |  |  | 100.00 |  | 100.00 | 100.00 |  | 100.00 |

**Fig. S5.** EDS spectra of a cleaned Cu thermally oxidized at 400 ºC electrode

**Table S5.** EDS data of a cleaned Cu electrode annealed at 400 ºC electrode.

| ***Element*** | ***Net***  ***Counts*** | ***Int.***  ***Cps/nA*** | ***Weight %*** | ***Weight %***  ***Error*** | ***Norm.***  ***Wt.%*** | ***Atom %*** | ***Atom %***  ***Error*** | ***Compnd %*** |
| --- | --- | --- | --- | --- | --- | --- | --- | --- |
| ***C*** | 149 | --- | 1.16 | ± 0.15 | 1.16 | 3.99 | ± 0.51 | 1.16 |
| ***O*** | 3337 | --- | 16.32 | ± 0.31 | 16.32 | 42.23 | ± 0.80 | 16.32 |
| ***Cu*** | 4250 | --- | 82.53 | ± 2.50 | 82.53 | 53.78 | ± 1.63 | 82.53 |
| ***Cu*** | 15921 | --- | --- | --- | --- | --- | --- | --- |
| ***Total*** |  |  | 100.00 |  | 100.00 | 100.00 |  | 100.00 |


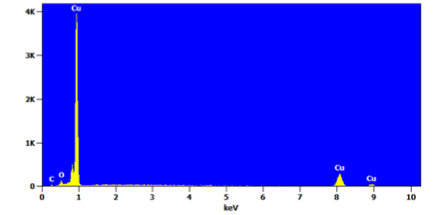


**Fig. S6** EDS spectra of a rCu electrode.

**Table S6.** EDS data of rCu electrode.

| ***Element*** | ***Net***  ***Counts*** | ***Int.***  ***Cps/nA*** | ***Weight %*** | ***Weight %***  ***Error*** | ***Norm.***  ***Wt.%*** | ***Atom %*** | ***Atom %***  ***Error*** | ***Compnd %*** |
| --- | --- | --- | --- | --- | --- | --- | --- | --- |
| ***C*** | 182 | --- | 1.54 | ± 0.16 | 1.54 | 7.10 | ± 0.74 | 1.54 |
| ***O*** | 498 | --- | 2.68 | ± 0.25 | 2.68 | 9.30 | ± 0.88 | 2.68 |
| ***Cu*** | 4947 | --- | 95.78 | ± 2.69 | 95.78 | 83.60 | ± 2.35 | 95.78 |
| ***Cu*** | 28171 | --- | --- | --- | --- | --- | --- | --- |
| ***Total*** |  |  | 100.00 |  | 100.00 | 100.00 |  | 100.00 |

Tafel slopes were estimated by fitting polarization data to equation, η = *b**log(*j*) + *a*, where η is the overpotential, *b* is the Tafel slope, *j* is the current density, and *a* is a constant.

**Table S7.** Tafel slopes of Ag, rAg, Cu and rCu electrodes.

| **Electrode** | **Tafel slope (mV/dec)** |
| --- | --- |
| Ag | 289.33 |
| rAG | 192.7 |
| Cu | 431.26 |
| rCu | 350.77 |


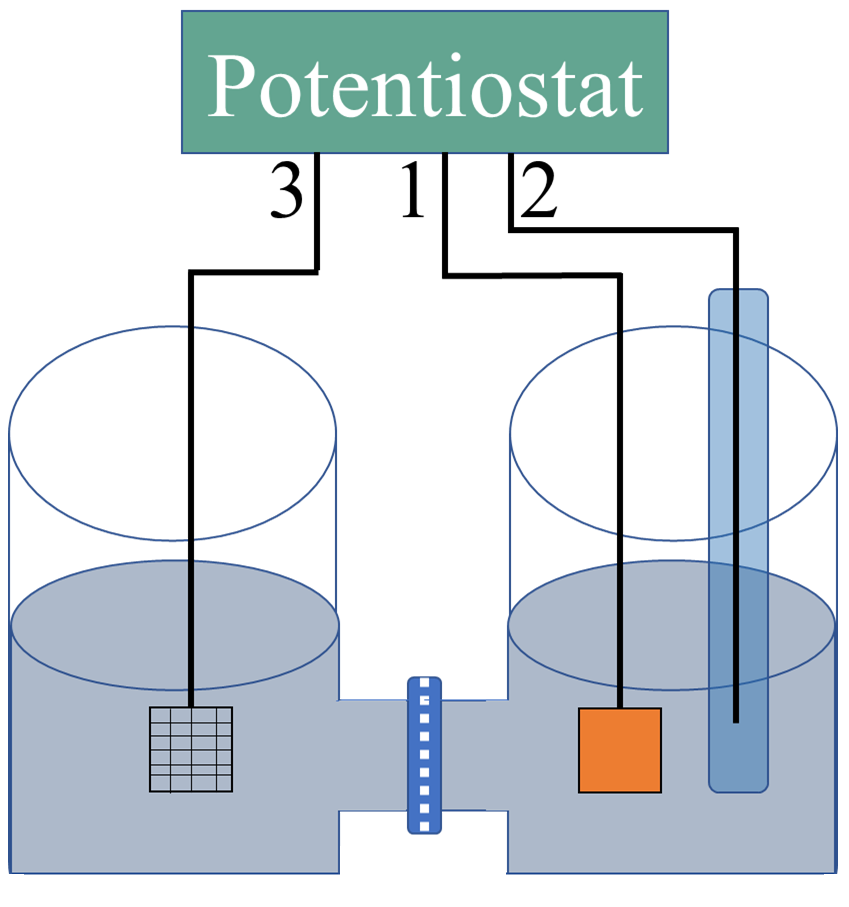


**Fig. S7** Schematic of two-compartment three terminal electrochemical cell (1. Working electrode, 2. Ag/AgCl reference electrode saturated in KCl and 3. Pt mesh counter electrode).

**Table S8.** Performance comparison of similar catalysts under the same electrolyte.

| **Catalyst** | **Electrolyte** | **Current density @ -0.5 V vs. RHE** | **Ref** |
| --- | --- | --- | --- |
| Ag foil | 0.5 M borate buffer (pH 9.2) | 1 | [1] |
| OD-Ag | 0.5 M borate buffer (pH 9.2) | 3.13 | [1] |
| Cu foil | 0.5 M borate buffer (pH 9.2) | 1.75 | [2] |
| Sputter deposited Ag | 0.5 M borate buffer (pH 9.2) | 1.6 | [2] |
| Electrodeposited Ag | 0.5 M borate buffer (pH 9.2) | 1.25 | [2] |
| Ag | 0.5 M borate buffer (pH 9.2) | 2.9 | This work |
| rAg | 0.5 M borate buffer (pH 9.2) | 10.01 | This work |
| Cu | 0.5 M borate buffer (pH 9.2) | 1 | This work |
| rCu | 0.5 M borate buffer (pH 9.2) | 1.98 | This work |

**Table S9.** BHMF concentration and yield are estimated from constant potential electrolysis (CPE) data.

| **Electrode** | **Potential (V vs. RHE)** | **Current (mA/cm^2^)** | **Time (s)** | **Charge (C)** | **Number of electrons (x 10^19^)** | **Number of BHMF molecules (x 10^19^)** | **BHMF (mM)** | **Yield %** |
| --- | --- | --- | --- | --- | --- | --- | --- | --- |
| rAg | -0.5 | 9.7 | 1800 | 17.46 | 10.91 | 5.46 | 4.53 | 22.65 |
| rAg | -0.6 | 12.8 | 1800 | 23.04 | 14.4 | 7.2 | 5.98 | 29.89 |
| rAg | -0.7 | 15.5 | 1800 | 27.90 | 17.44 | 8.72 | 7.24 | 36.19 |
| rCu | -0.5 | 1.96 | 1800 | 3.53 | 2.21 | 1.11 | 0.92 | 4.58 |
| rCu | -0.6 | 2.7 | 1800 | 4.86 | 3.04 | 1.52 | 1.26 | 6.30 |
| rCu | -0.7 | 3.65 | 1800 | 6.57 | 4.11 | 2.05 | 1.70 | 8.52 |

Relative concentration is determined from NMR data by using the following equation,

M_x_/M_y_ = (I_x_/I_y_)*(N_y_/N_x_),

Where I is integral and N is number of nuclei giving rise to signal M_x_/M_y_ is molar ratio of the two compounds. BHMF concentration is increasing with the increase in applied potential which is in agreement with the BHMF concentration estimated from electrolysis data. The minor discrepancy observed in the BHMF concentration between the two methods may be attributed to experimental uncertainties.

**Table S10.** BHMF concentration is estimated from NMR data.

| **Electrode** | **Potential (V vs. RHE)** | **I_x_** | **I_y_** | **N_x_** | **N_y_** | **HMF conc with 10% D_2_O (M)** | **BHMF concentration (mM)** |
| --- | --- | --- | --- | --- | --- | --- | --- |
| rAg | -0.5 | 1.1446 | 0.6133 | 1 | 2 | 0.018 | 4.8 |
| rAg | -0.6 | 1.1447 | 0.6221 | 1 | 2 | 0.018 | 4.9 |
| rAg | -0.7 | 1.1454 | 0.8015 | 1 | 2 | 0.018 | 6.3 |
| rCu | -0.5 | 1.1454 | 0.1822 | 1 | 2 | 0.018 | 1.4 |
| rCu | -0.6 | 1.1454 | 0.1837 | 1 | 2 | 0.018 | 1.4 |
| rCu | -0.7 | 1.1454 | 0.3107 | 1 | 2 | 0.018 | 2.4 |

**References:**

[1] H. Liu, T.-H. Lee, Y. Chen, E.W. Cochran, and W. Li, Paired electrolysis of 5-(hydroxymethyl)furfural in flow cells with a high-performance oxide-derived silver cathode. Green Chemistry 23 (2021) 5056-5063.

[2] J.J. Roylance, T.W. Kim, and K.-S. Choi, Efficient and Selective Electrochemical and Photoelectrochemical Reduction of 5-Hydroxymethylfurfural to 2,5-Bis(hydroxymethyl)furan using Water as the Hydrogen Source. ACS Catalysis 6 (2016) 1840-1847.
